# In Vivo Positional Analysis of Implantable Collamer Lens Using Ultrasound Biomicroscopy

**DOI:** 10.1155/2016/4060467

**Published:** 2016-09-08

**Authors:** Ahmed Mohamed Kamal Elshafei, Mahmoud Mohamed Genaidy, Hossam Mohamed Moharram

**Affiliations:** Ophthalmology Department, Minia University, Minia, Egypt

## Abstract

*Purpose*. To evaluate the anterior segment, the anatomical position of the implantable collamer lenses (ICL), and its relationship to adjacent ocular structures using Ultrasound Biomicroscopy (UBM).* Methods*. In a prospective study, 142 myopic eyes of 93 patients implanted with Visian ICL were subjected to UBM examination between March 2010 and January 2015. The relative position of ICL to the adjacent structure and the overall iris configuration were evaluated. The machine calibers were used to measure the minimum central distance between the ICL and anterior lens capsule (vault) and the vertical central distance between the corneal endothelium and the ICL (E-ICL).* Results*. The mean ICL vault was 376 ± 105 *μ*m. The mean E-ICL was 2826 ± 331 *μ*m. Contact between ICL and the posterior epithelium of the iris was present in all eyes. The overall iris configuration was flat in 89 eyes. Central anterior convexity was present in 41 eyes and mild peripheral iris bombe in 12 eyes. The haptics could be imaged in the ciliary sulcus in 112 eyes and at least one haptic resting on the lens periphery and zonules in 30 eyes.* Conclusion*. UBM can provide valuable anatomical information that allows detailed postoperative in vivo assessment of ICL.

## 1. Introduction

The implantable collamer lens (ICL) is a flexible, posterior chamber phakic intraocular lens [1]. It is implanted in the ciliary sulcus and is vaulted to avoid contact with the crystalline lens. ICL implantation is an effective option for the treatment of refractive errors, offering an optical quality superior to that of corneal refractive surgeries [2]. According to data from the ICL in Treatment of Myopia Study Group [3], ICL implantation is a safe and effective option for patients with moderate to high myopia. The ICL's designs and materials were refined in several clinical studies through a series of prototypes. Recent advances in ophthalmic anterior segment imaging such as Scheimpflug photography, very high-frequency ultrasonography, and anterior segment optical coherence tomography (AS-OCT) have been used to evaluate the position and vault of posterior chamber phakic intraocular lens and their dynamics with intraocular changes [4–6].

Ultrasound Biomicroscopy (UBM) is a high-frequency ultrasound technology that can provide in vivo high resolution (near microscopic resolution) cross-sectional images of anterior segment and objective quantitative measurements of anterior segment parameters in living patients [7, 8]. It provides a unique tool to noninvasively evaluate the relations of these implants within the posterior chamber and helps to analyze the mechanisms of crystalline lens and iris complications [9]. However, it is a contact technique that requires topical anesthesia and an immersion bath with a coupling medium with a risk of infection or corneal injury. Noncontact imaging devices including AS-OCT and Scheimpflug photography are fast and easy for the examiner and patient and are especially better for examining patients in early postoperative period. However, these devices have an important limitation that they cannot measure any object behind opaque structure. On the other hand, UBM can obtain clear images through opaque structures including the iris and allows visualization of the ciliary sulcus [10].

The purpose is to evaluate the anterior segment, the anatomical position of ICL, and its relationship to adjacent ocular structures using UBM.

## 2. Patients and Methods

### 2.1. Design: Prospective Clinical Study

142 eyes of 93 patients implanted with spherical Visian ICL (model V4 STAAR Surgical AG, Nidau, Switzerland) for correction of myopia were enrolled in this study. The patients were 57 females (61.3%) and 36 males (38.7%). The mean age was 24.1 years ±5 (range 16–37 years).

Preoperative evaluation of the patients was done using Pentacam*™* (Oculus) to measure white to white diameter (*W*-*W*), keratometric (*K*) readings, and internal anterior chamber depth (ACD) from the corneal endothelium to the crystalline lens.

Although UBM is the most accurate method for preoperative determination of *W*-*W*, this requires a 40 MHz machine that allows full sulcus to sulcus measurement in one image. Unfortunately, the machine used in this study is a 50 MHz one that provides better resolution but smaller angular field. Pentacam was used to measure *W*-*W* as it is more objective than caliper measurement and the ICL diameter was ordered accordingly.

All surgeries were done under general anesthesia through temporal clear corneal incision. Intraoperative peripheral iridectomy using 23G vitreous cutter was done after lens implantation. This was followed by viscoelastic removal and wound hydration. Postoperative topical steroid and antibiotics were used for one month. Topical ocular hypotensive was used in the early postoperative period if the intraocular pressure was elevated.

Postoperative clinical examination included slit-lamp biomicroscopic examination of the anterior segment to evaluate the ICL position, patency of iridotomies, iris configuration, and clarity of the crystalline lens. Other examinations included measurement of the intraocular pressure (IOP), postoperative refraction, and uncorrected visual acuity (UCVA).

UBM study was done for all patients in the Ophthalmology Department of Minia University Hospital using immersion technique with Paradigm*™* UBM six months after ICL implantation between March 2010 and January 2015.

UBM examination technique included

**Figure 1:**
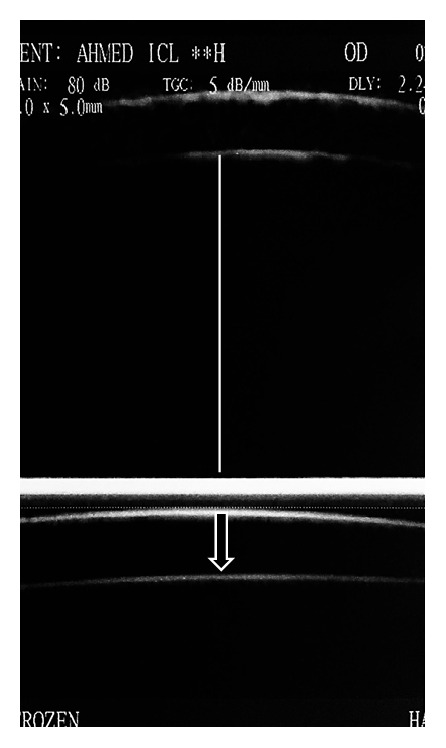
Axial scanning through the center of the cornea, anterior chamber, center of the pupil, ICL, and anterior lens capsule. The image shows endothelium to ICL distance (line) and ICL vault (arrow).

**Figure 2:**
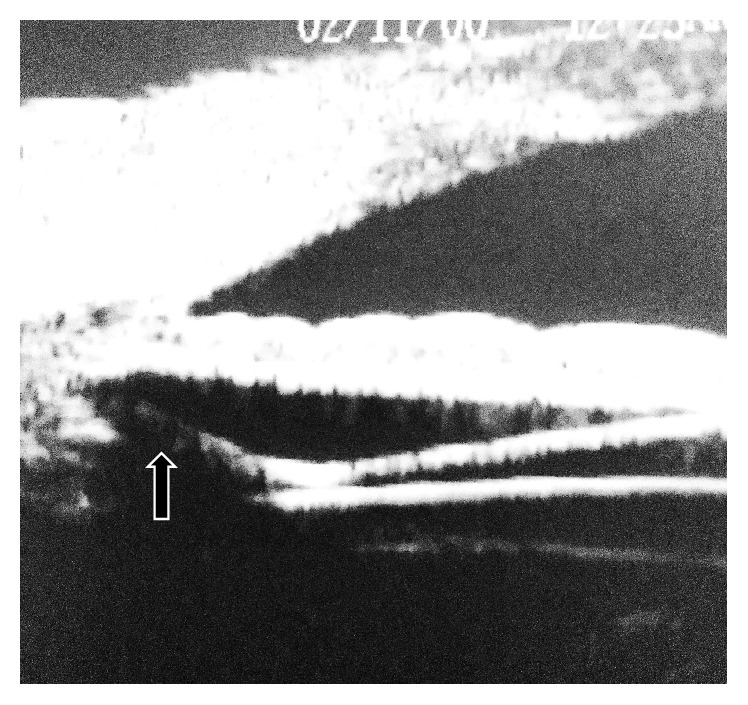
Radial scanning through the angle of the anterior chamber, iris, posterior chamber, ICL haptics, and ciliary body. The haptic rests in the ciliary sulcus (arrow).

1. axial scanning through the center of the cornea, anterior chamber, center of the pupil, ICL, and anterior lens capsule (Figure 1),
2. radial scanning in the four quadrants to image the angle of the anterior chamber, iris, posterior chamber, ICL haptics, and ciliary body (Figure 2).

UBM examination was done under the same ordinary room lighting condition and while the patient was fixating on the ceiling to alleviate the effect of illumination and accommodation on the anatomical relations and measurements.

The UBM machine calibers were used to measure the minimum central distance between the ICL and anterior lens capsule (vault) and the vertical central distance between the corneal endothelium and the ICL (E-ICL). Well centered high resolution axial images were used for measurement.

Using radial scans, the relation between ICL and the posterior surface of the iris and the position of ICL haptics together with the overall iris configuration were evaluated. Scans were taken in upper nasal, upper temporal, lower nasal, and lower temporal quadrants where the haptics of ICL were suspected to rest.

This study was approved by the local ethics committee and adhered to the tenets of the Declaration of Helsinki. All patients agreed to be enrolled in the study following a thorough explanation of the purpose of the study and the methodology used.

### 2.2. Statistical Analysis

Data were analyzed using SPSS Version 20.0 for Windows (Statistical Package for the Social Sciences). Quantitative data were presented by mean and standard deviation, while qualitative data were presented by frequency distribution. The associations between continuous variables were determined using Pearson Product-Moment Correlation. Kolmogorov-Smirnov test was applied for all variables and resulted in nonsignificant outcomes indicating the normality of data distribution. The probability of less than 0.05 was used as a cut-off point for all significant tests.

## 3. Results

### 3.1. Preoperative Data (Table 1)

The mean preoperative spherical error of refraction was −14.1 ± 4.4 diopters (D) and the mean cylindrical error was 2.3 ± 1.2 D. The mean white to white diameter was 11.6 ± 0.6 mm (range 11 to 13 mm). Keratometric readings ranged from 39 to 48.25 D with a mean of 43.2 ± 1.9 D for *K*1 and from 40 to 51.6 D with a mean of 44.6 ± 2.1 D for *K*2. The mean central corneal thickness was 519 ± .043 *μ*m and the mean internal ACD was 3200 ± 0.27 *μ*m. The mean power of implanted ICL was −15.5 ± 3.7 D and the mean implant diameter was 12.6 ± 0.5 mm (Table 2).

### 3.2. Postoperative Data

**Figure 3:**
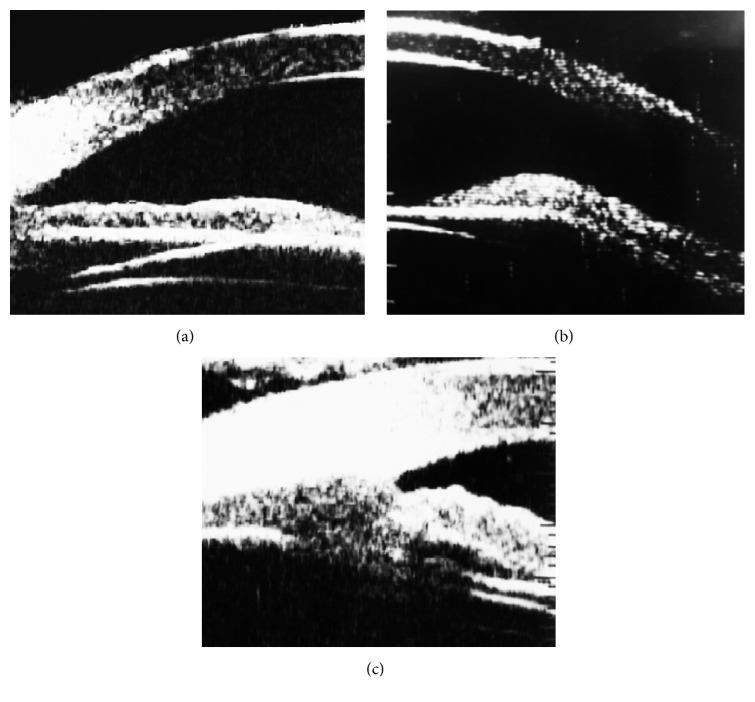
Overall iris configuration. (a) Flat iris; (b) central anterior convexity; (c) peripheral iris bombe.

**Figure 4:**
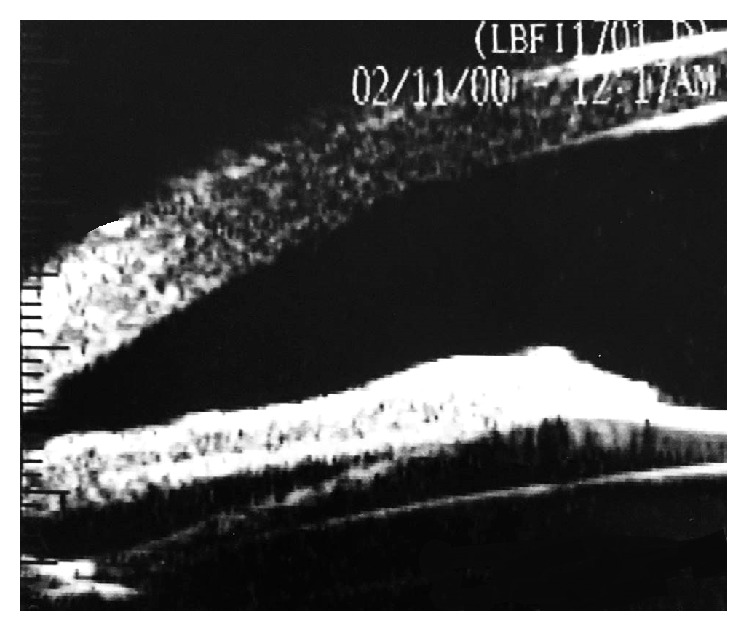
Contact between ICL and the posterior surface of the iris.

**Figure 5:**
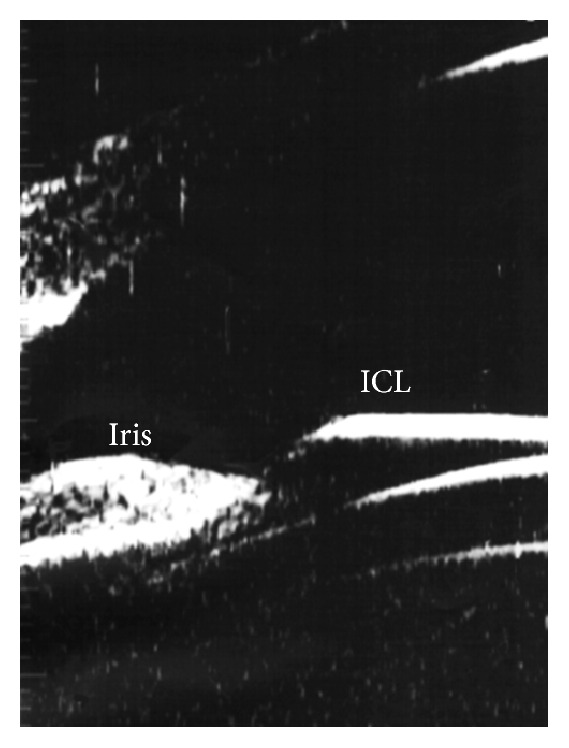
Anterior displacement of the optic and optic haptic junction.

The mean ICL vault was 376 ± 105 *μ*m (range 192 to 402 *μ*m). The mean E-ICL was 2826 ± 331 *μ*m (range 2159 to 3079 *μ*m). The lens haptics could be imaged resting in the ciliary sulcus (Figure 2) in 112 eyes (78.87%) and at least one haptic rested on the lens periphery and zonules in 30 eyes (21.12%). The overall iris configuration (Figure 3) was flat in 89 eyes (62.76%) with central anterior convexity in at least one quadrant in 41 eyes (28.87%) and peripheral iris bombe in at least one quadrant in 12 eyes (8.45%). Contact between ICL and the posterior surface of the iris was present in all eyes (Figure 4). This contact was limited to the central third of the iris in 78 eyes and extended more peripherally in 64 eyes. One eye developed persistent increase in the IOP that necessitated the use of topical antiglaucoma medications. On UBM imaging there was marked forward displacement of the optic and optic haptic junction (Figure 5) with very narrow angle. Posterior iris cyst was imaged in one eye. Cataract developed in three eyes. In two eyes the vault was less than 200 *μ*m while in the third one the vault was 264 *μ*m.

There was a high statistically significant strong correlation between internal ACD and the summation of E-ICL and vault (*P* < 0.001, *r* = 0.78). There was a high statistically significant fair correlation between vault and the preoperative internal ACD (*P* = 0.001, *r* = 0.28) and a statistically significant fair correlation between vault and preoperative spherical error (*P* = 0.01, *r* = 0.25). Nonsignificant week correlation was found between vault and *W*-*W* (*P* = 0.8, *r* = 0.02).

## 4. Discussion 

In this study the 50 MHz Paradigm UBM was used for in vivo evaluation of Visian 4 ICL. The biologic nature of the ICL's soft collamer material allows good quality imaging unlike the PMMA IOLs that cause many reflections. The high-frequency ultrasound can penetrate the iris and visualize the sulcus and the lens within the posterior chamber. UBM is done in supine position and requires a plastic or silicone eyecup to hold a coupling medium (methyl cellulose or saline solution) and this can induce pressure on the eyeball and may influence the results [10]. However, it has long been known that the immersion technique is more accurate than direct contact technique which may exert more pressure [11].

UBM examination is a relatively difficult procedure and needs experienced examiner. It causes some patient inconvenience so it is difficult to be repeated. It could not be performed in the early postoperative period for fear of infection.

Central vault is very important factor for the postoperative safety of ICL surgery. Insufficient vault can lead to cataract formation while excessive vault may induce glaucoma [12]. Previous studies defined an excellent vault as 250 to 750 *μ*m [13]. In this study the mean vault was 376 ± 105 *μ*m and the mean E-ICL was 2826 ± 331 *μ*m. Several previous studies used anterior segment imaging devices to measure vault and E-ICL with relatively comparable results. Using UBM, Pitault et al., [9] found that the mean central vault was 402 ± 194 *μ*m and the mean distance between ICL and the central endothelium was 2398 ± 203 *μ*m.

Zhang et al. [10] used both UBM and anterior segment OCT in evaluation of cornea to ICL and central vault measurement in myopic eyes implanted with Visian ICL. They found that the mean central vault was 440 ± 190 *μ*m and E-ICL distance was 2490 ± 250 *μ*m when measured by UBM. Vault measured with OCT was significantly higher than that obtained with the UBM while the cornea to ICL distance measured with two devices was not statistically significantly different. In the current study UBM examinations were done in the ordinary room lighting condition while, in the study of Zhang et al., the examinations were performed in a dark room with ambient illumination below 5 lx.

Du et al. [14] used UBM to study the changes in ACD and the vaulting between the Visian ICL and the crystalline lens during pharmacologic accommodation (with topical pilocarpine). They found that there was a significant decrease in vault accompanied by a significant increase in E-ICL distance after instillation of pilocarpine (*P* < 0.01). They concluded that, during pharmacologic accommodation, the ICL and the crystalline lens came closer as the ICL was pushed backward by the iris as a result of pupillary constriction. Simultaneously, the anterior surface of the crystalline lens became more convex and moved forward.

Lindland et al. [15] studied the relationship between vaulting and anterior subcapsular opacification. They found contact between the ICL and the crystalline lens in 15.6% of the eyes and anterior subcapsular opacification developed in 13.0% of eyes. Compared to the previous study, our results showed significantly less percentage of eyes that developed cataract. Only three eyes (2.11%) had cataract. The vault was less than 200 *μ*m in two eyes while in the third eye the vault was 264 *μ*m. No direct contact was present in the three eyes. Although decreased vault is an important risk factor for cataract formation, other factors including operative trauma, postoperative inflammation, and myopia itself may have a role.

Most of the previous similar studies focused on the relation between ICL and the anterior surface of the crystalline lens. The classic concept is that undersizing of ICL leads to small vault and hence the increased incidence of cataract formation and oversizing of ICL; on the other hand, increased vault will push the iris forward increasing the risk of decreased angle width and glaucoma [10]. However, previous studies did not focus on the relation between ICL and the posterior surface of the iris. In the current study we found contact between ICL and the posterior epithelium of the iris in all examined eyes. This contact was unrelated to increased vault. Such contact theoretically increases the risk of pigment dispersion from the posterior surface of the iris especially after physical exercise or accommodation like what happens in pigment dispersion syndrome. Long term follow-up of the IOP, corneal endothelium, and angle pigmentation is recommended to prove or to contradict this possibility.

## 5. Conclusion

UBM can provide valuable anatomical information that allows detailed postoperative in vivo assessment of ICL.

## Figures and Tables

**Table 1:** Preoperative eye measurements.

Variable	Range	Mean ± SD
Spherical	−5.5–−24 D	−14.1 ± 4.4 D
Cylindrical	0.0–−3.5 D	2.3 ± 1.2 D
Axis	5°–180°	100.2 ± 60.4°

Axial length	24.4–32.97 mm	28.5 ± 1.6 mm

White to white	11–13 mm	11.6 ± 0.6 mm

*K*1	39–48.25 D	43.2 ± 1.9 D

*K*2	40–51.6 D	44.6 ± 2.1 D

ACD	2.8–4.12 mm	3.2 ± 0.27 mm

**Table 2:** ICL characteristics.

Variable	Range	Mean ± SD
ICL power	−6–−23 D	15.5 ± 3.7 D
ICL diameter	12-13 mm	12.6 ± 0.5 D

## References

[1] Arne J. L., Lesueur L. C. (2000). Phakic posterior chamber lenses for high myopia: functional and anatomical outcomes. *Journal of Cataract and Refractive Surgery*.

[2] Kamiya K., Igarashi A., Shimizu K., Matsumura K., Komatsu M. (2012). Visual performance after posterior chamber phakic intraocular lens implantation and wavefront-guided laser in situ keratomileusis for low to moderate myopia. *American Journal of Ophthalmology*.

[3] ICL in Treatment of Myopia (ITM) Study Group (2004). United States Food and Drug Administration clinical trial of the Implantable Collamer Lens (ICL) for moderate to high myopia: three-year follow-up. *Ophthalmology*.

[4] Alió J. L., Piñero D. P., Sala E., Amparo F. (2010). Intraocular stability of an angle-supported phakic intraocular lens with changes in pupil diameter. *Journal of Cataract and Refractive Surgery*.

[5] Konstantopoulos A., Hossain P., Anderson D. F. (2007). Recent advances in ophthalmic anterior segment imaging: a new era for ophthalmic diagnosis?. *British Journal of Ophthalmology*.

[6] Baïkoff G. (2006). Anterior segment OCT and phakic intraocular lenses: a perspective. *Journal of Cataract and Refractive Surgery*.

[7] Pavlin C. J., Foster F. S. (1995). *Ultrasound Biomicroscopy of the Eye*.

[8] Kojima T., Yokoyama S., Ito M. (2012). Optimization of an implantable collamer lens sizing method using high-frequency ultrasound biomicroscopy. *American Journal of Ophthalmology*.

[9] Pitault G., Leboeuf C., Leroux Les Jardins S., Auclin F., Chong-Sit D., Baudouin C. (2005). Ultrasound biomicroscopy of posterior chamber phakic intraocular lenses: a comparative study between ICL and PRL models. *Journal Francais d'Ophtalmologie*.

[10] Zhang J., Luo H.-H., Zhuang J., Yu K.-M. (2016). Comparison of anterior section parameters using anterior segment optical coherence tomography and ultrasound biomicroscopy in myopic patients after ICL implantation. *International Journal of Ophthalmology*.

[11] Trivedi R. H., Wilson M. E. (2011). Axial length measurements by contact and immersion techniques in pediatric eyes with cataract. *Ophthalmology*.

[12] Fernandes P., González-Méijome J. M., Madrid-Costa D., Ferrer-Blasco T., Jorge J., Montés-Micó R. (2011). Implantable collamer posterior chamber intraocular lenses: a review of potential complications. *Journal of Refractive Surgery*.

[13] Kamiya K., Shimizu K., Komatsu M. (2009). Factors affecting vaulting after implantable collamer lens implantation. *Journal of Cataract & Refractive Surgery*.

[14] Du C., Wang J., Wang X., Dong Y., Gu Y., Shen Y. (2012). Ultrasound biomicroscopy of anterior segment accommodative changes with posterior chamber phakic intraocular lens in high myopia. *Ophthalmology*.

[15] Lindland A., Heger H., Kugelberg M., Zetterström C. (2010). Vaulting of myopic and toric implantable collamer lenses during accommodation measured with visante optical coherence tomography. *Ophthalmology*.

